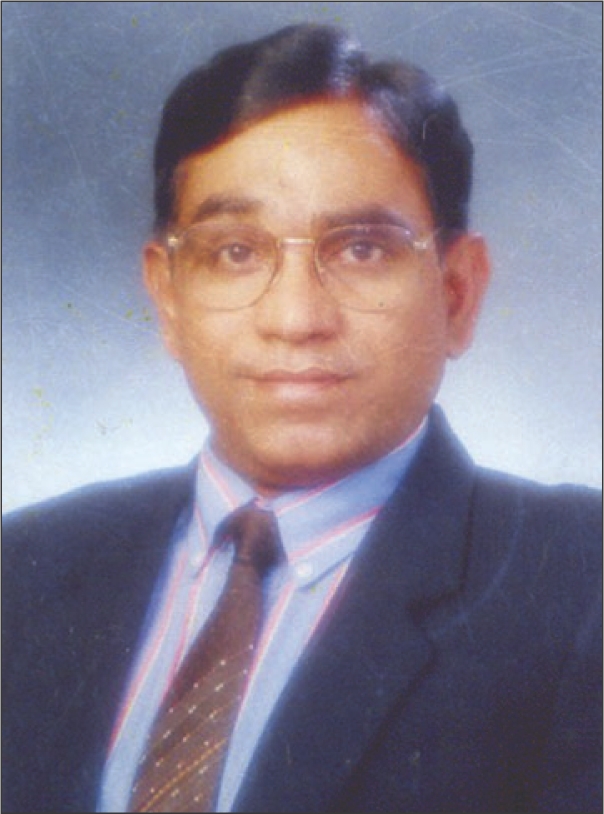# Presidential address

**DOI:** 10.4103/0971-3026.45335

**Published:** 2009-02

**Authors:** K Prabhakar Reddy

**Affiliations:** President IRIA, Plot No. 481/A, Jubilee Heights, Road No. 86, Phase-3, Jubilee Hills, Hyderabad - 500 033, India E-mail: drkundur@yahoo.co.in

At the outset, I would like to extend my hearty greetings and best wishes for the year 2009, with the hope that this year will usher in prosperity, and I warmly welcome you all to the 62^nd^ Annual Conference of the IRIA, being hosted in this beautiful historical city of Patna.

I am indeed honored to be addressing this August gathering, as President of the “Indian Radiological and Imaging Association”. My journey during these years has been arduous but very challenging and I thank each one of you for providing me this opportunity to hold several posts and electing me as the President of our sacred organization.

Today in the context of health care, Radiology has earned the distinction and reputation of being an important specialty in diagnosis and treatment. With the explosion of health care facilities, the opportunities for Radiologists have increased many-fold. I am delighted that our young Radiologists have risen to such heights and have done our country proud.

At present, we are a strong family of over 7000 members and outside USA, the “Indian Radiological and Imaging Association” is considered one of the largest forums in radiology. The contribution of many of our colleagues' world over is remarkable.

In order to sustain this tempo of achieving excellence, I have a vision and a mission to take the benefits of Imaging and Radiology to the nook and corner of our country. If we have to fulfill the objective, then we have to collectively focus in promoting knowledge to our younger generation. At this juncture I am pleased to convey that from this year we have started to financially support individual state chapters for conducting CME programs. The IRIA will be conducting Resident Teaching Programs in various cities. More importantly, to encourage more participation in International conferences, such as those organized by the Radiology Society of North America (RSNA), Asia-Oceanian Society of Radiology (AOSR), International Society of Radiology (ISR) and South Asian Association for Regional Cooperation (SAARC), IRIA has started providing financial aid to our members.

I request the Medical Council of India (MCI) to sanction accreditation for National Conferences and the CME programs. I also urge MCI to include a uniform syllabus for MBBS and MD in the field of Radiology.

Apart from the above, for rapid dissemination of knowledge, all the teaching hospitals in the country must network with each other under the auspicious of the University Grants Commission to promote a knowledge hub. In this context, I am keen that our academic body, the Indian College of Radiology and Imaging (ICRI) should be more proactive and help teaching institutions to achieve this objective.

In the recent past, we have witnessed an enormous increase in the cost of providing proper health care, and in this context I implore the Central Government to offer relief in the form of tax concessions for equipments and drugs, which should benefit the poorest of the poor. The industry must also share the burden of serving the unprivileged poor. What is required is handholding between IRIA and the industry.

I also urge the Government to increase the quota of seats in the MD and DNB courses, which is the need of the hour.

During my presidentship I would strive to ensure that the Pre-Conception and Pre Natal Sex Determination (PC and PNDT) Act should be implemented without fear or favor and programs on “Save the Girl Child” should be popularized by our colleagues. In the same breath I would like to place on record that over zealous Government functionaries have unnecessarily harassed many of our colleagues for no valid reasons. The IRIA should step-in to put a halt to this practice.

There is a tremendous impetus offered by the Central and State Governments in promoting the concept of public private participation in the field of Radiology. I have noticed that many states have outsourced the services of Radiologists in order to cover unexplored territories. I for one appreciate and acknowledge such a move and would encourage the Government to use the medium of teleradiology more effectively.

And for this to succeed the role of the trade and the technology partners cannot be underestimated. It is my earnest appeal to the captains of our industry to come forward and participate in such noble objectives by offering their expertise. This model will galvanize the health delivery system and will help in offering affordable treatment to the unprivileged.

The role of the Atomic Energy Regulartory Board (AERB) in regulating the use of X-Ray emitting equipments is laudable and I want to request them to conduct programs on the hazards of radiation to all the undergraduates in the country. The importance of this need not be over-emphasized.

My focus during my tenure would be to propose an insurance welfare program for all our members.

It will be my endeavor to restrict the practice of ultrasound only to Radiologists and any training program by any agency must have the approval of the IRIA.

The System of DNB and MD exams should be “Objective Structured Clinical Examination” (OSCE), which will ensure more transparency.

We should increase the membership enrolment by at least another thousand during this year.

On this momentous occasion, I wish to place on record my deep appreciation of the contribution of our senior members and past-Presidents and I assure this august gathering that I would carry on their legacy of compassion, of purpose and of hard work. With your support I will strive to uphold the dignity of this Presidentship and will carry the IRIA to lofty heights.

I believe in convergence of ideas and I am available day and night for any help.

Friends, I am indebted to everyone for helping me achieve this position and I wish to especially thank all my family members for their abounding sense of understanding and their support, which helped me to attain this status. My parents, my wife Aruna, and my children Dr. Prashanth and Dr. Sweta deserve special gratitude from me.

I would like to thank all my well-wishers, seniors, colleagues and members of the industry for their unstinted support throughout my tenure in the IRIA.

May God bless you all with happiness and good health!

Long live IRIA!

**Figure:**